# Characterizing Horizontal and Vertical Perspectives of Spatial Equity for Various Urban Green Spaces: A Case Study of Wuhan, China

**DOI:** 10.3389/fpubh.2020.00010

**Published:** 2020-02-19

**Authors:** Sanwei He, Yilin Wu, Lei Wang

**Affiliations:** ^1^Key Laboratory of Urban Land Resources Monitoring and Simulation, Ministry of Natural Resources, Shenzhen, China; ^2^School of Public Administration, Zhongnan University of Economics and Law, Wuhan, China; ^3^Key Laboratory of Watershed Geographic Sciences, Nanjing Institute of Geography and Limnology, Chinese Academy of Sciences, Nanjing, China; ^4^Department of Planning and Environmental Management, University of Manchester, Manchester, United Kingdom

**Keywords:** spatial equity, Theil index, green spaces, environmental justice, China

## Abstract

Equity has been a major concern of urban green space provision. Whether the urban green spaces are equitably provided for socially disadvantaged groups is an important issue in the field of social and environmental justice. This topic is particularly significant in fast-growing Asian countries like China experiencing widening income disparity. This paper examines whether and to what extent the different green spaces (including public parks and urban vegetation) are equitable for all populations (referring to horizontal equity) and also for different social groups (referring to vertical equity) in this typical inland city—Wuhan, China. A novel indicator combining proximity and quality is presented to assess the supply of public parks. The Theil index provides a decomposable measure of overall equity across different regions and vulnerable groups. Both horizontal and vertical perspectives are compared to characterize the spatial equity of urban green spaces (including public parks and urban vegetation) across all population and across different social groups. The empirical analysis of the inland city showed that the overall supply of public parks is far more unequal than mixed or woody vegetation. The distribution of public parks is more inequitable in the outer area, whereas the distribution of mixed or woody vegetation is more inequitable in the inner area. Furthermore, the geographic detector analysis is employed to investigate the spatial relation between socioeconomic contexts and urban green spaces. The spatial heterogeneity of education and age groups is statistically significant for explaining the distribution of public parks. Meanwhile, population density clearly plays a role in the distribution of both public parks and urban vegetation. Per capita income can explain 26% of the distribution of public parks but is not significantly associated with mixed or woody vegetation. Finally, the vertical equity of urban green space is also examined in this paper that the vulnerable groups in the inner area, such as females, residents with low education, children, and the elder suffer from highly unequal accessibility to parks, whereas the vulnerable group in the outer area, such as the migrants gets unequal access to parks.

## Introduction

As providers of regulating and cultural ecosystem services, the significance of green spaces has been widely acknowledged, such as purifying the air, reducing traffic noise pollution, increasing carbon storage capacity, reducing urban energy consumption, promoting the physiological and mental health of urban residents, etc. (1, 2). Because of these social, economic, and environmental benefits, there may be competition in societies' interactions with urban green space, leading to the equity issue to determine the fairness of such interactions (3). With increasing urbanization in the globe especially in Asian countries, the issue of population densification in urban areas puts much pressure on maintaining and improving the amount and quality of green space supply (4). Sustainable urban development requires supply and demand mismatches of green space are to be reconciled and the needs of different stakeholders are to be balanced (5).

Horizontal and vertical equity were initially defined for evaluating tax reform (6). The horizontal equity as a minimal rule of fairness requires the equal treatment of all population whereas the vertical equity calls for an appropriate pattern of differentiation (inequality in treatment) among people who are not equals, such as the low-income, children, or ethnic minorities (7). Such a concern raises very difficult problems when comparing groups across different cultures or even different social classes as almost inevitably follows when dealing with vertical equity (8). There is common support for improving horizontal equity while remaining neutral on the controversial issue of vertical equity.

There is a small but growing body of studies in U.S. or Europe exploring the differentiated distribution of green space in relation to age, religion ethnicity, minority status, and education. Taking 10 US urbanized areas as examples, Nesbitt et al. (9) find a strong positive correlation between urban vegetation and higher education and income across most cities, and negative correlations between racial minority status and urban vegetation. Jay and Schraml (10) focus on immigrants and examine the role of urban forests for migrants in terms of their uses, perception, and integrative potential. La Rosa et al. (11) presents a planning framework for urban green spaces that aims to minimize social inequalities in green space accessibility and meet demands from social groups (e.g., children and elderly people).

These studies have started to emphasize the demand of socially disadvantaged groups for green spaces, but issues of equity and justice in the distribution and enjoyment of urban green spaces are still required, especially in the Chinese context. First, China is experiencing widening income disparity and urban gentrification is spreading across the continent (12). High-income people tend to enjoy the benefits of the environmental good. Second, the rural-to-urban migration is a quintessential feature of economic development and modernization in China. Almost 20% of the Chinese population are composed of migrants and their descendants. Influenced by the rigid *hukou* registration system, residents with migrant background are more likely to struggle with unemployment and low income (13). Uncovering the spatial equity of urban green spaces especially for different social groups is the basis to maximize societal benefits for the local government.

The distribution and governance of urban green space are inequitable in many cities around the world (14, 15). Public parks and woody vegetation are more often located in wealthy neighborhoods, where residents have higher education and income (16, 17). There is evidence that socioeconomically disadvantaged and racialized minorities have lower access to urban green space and are less likely to engage in urban forestry decision making (11, 15). It is important that residents have equitable opportunities to reach urban green space because of the ecosystem services that influence the well-being of residents, especially among the people who have lower political, social, and economic power.

Presently, the studies about social and environmental justice are mainly based on empirical studies of individual cities or regions in North America or Europe. There is evidence that variable results are produced among different geographical areas, different cultures, and urban areas with different development histories (10). The empirical study on the equity issue of urban green space in Chinese cities may consolidate the related theoretical framework in the field of environmental justice or political ecology and provide comparative results to the existing studies (18–20). It needs to be further explored how the equity of various urban green spaces differs across all population and across different social groups, and how the equity issue is associated with the spatiality of urban space.

This paper aims to provide horizontal and vertical perspectives for analyzing spatial equity of green space with a case study of Wuhan, using techniques, such as Theil index and geographic detector analysis. Then, the study area and methodology are described. Based on the overall horizontal inequality of green space supply, we decomposed the horizontal green space inequality according to the spatial structure into inequalities among the inner, middle, and outer areas. Furthermore, we mapped the horizontal equity gaps and observed the sweet spot and the sour spot of urban green spaces. Based on geographic detector analysis, the spatial relation between socioeconomic contexts and urban green spaces is investigated. The vertical equity is discussed as well to assess how the equity of various urban green spaces differs across different social groups. Finally, this paper ends with major conclusions and policy implications.

## Data Description and Methodology

### Data Description

#### The Study Area

Wuhan, with a longitude of 11341'11505′ and a latitude of 2958′3122′, is situated in the eastern part of Hubei in central China. By the end of 2017, Wuhan has a land area of 8,569.15 *km*^2^ with an urbanized area of 628 *km*^2^. The city is divided by the Yangtze River and Han River into three towns: the commercial town of Hankou, the industrial town of Hanyang, and the educational and high-tech town of Wuchang. The city's three ring roads demarcate three concentric areas of development: the inner area, the middle area, and the outer area.

In 2017, the total investment for city greening reaches 9.20 billion RMB and 659.35 hectares of green space areas were newly built this year. At the end of 2017, the areas of green space amounts to 20,947 hectares in the urbanized area of Wuhan and the coverage ratio of green spaces reaches 33.35%.

This paper chooses the largest inland city—Wuhan—as the study area for the following reasons. First, previous studies mainly focused on developed countries in North America or Europe. There are only a few empirical studies focusing on the issue of environmental justice in developing countries like China, but many of them paid much attention to coastal metropolitan cities like Beijing, Shanghai, and Shenzhen (21–23). The case study of inland cities helps to enrich the existing empirical findings (24). Second, Wuhan is undergoing rapid economic growth with a nominal growth rate of GDP in 2018 arriving at 10.72%. As the household income increases, residents in Wuhan raise new estimates of the demand for green space to enhance their quality of life. An assessment of whether the distribution of green space provides equitable opportunity for all residents, especially for disadvantaged social groups, such as low-income people, migrants, and children, is crucial for determining where such demands are best met. There are 2.57 million migrants living in Wuhan, accounting for almost 30% of all population. How they get access to urban green spaces and enjoy the environmental good are important for urban planners and policy-makers to improve social justice. Third, Wuhan is partitioned by the ring roads into three regions, namely, the inner, middle, and outer areas. It remains unknown how the spatial circular structure will have an influence on the spatial equity of various green spaces.

**Figure 1:**
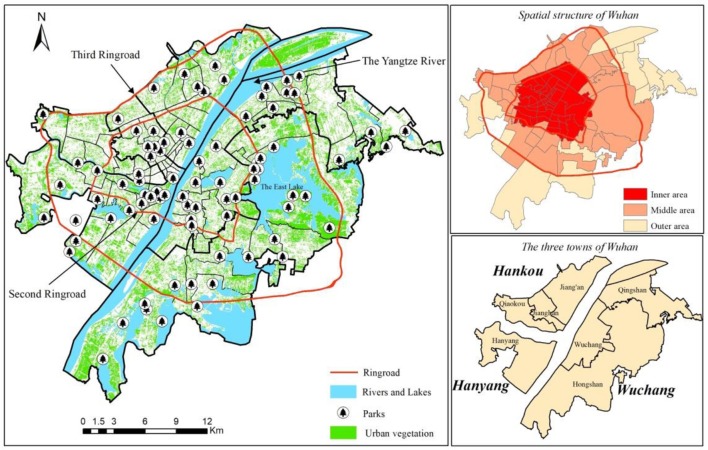
The study area of Wuhan.

The study area is composed of 89 *jiedao-level* sub-districts in seven urban districts of Wuhan as seen in Figure 1. The population density of the study area is 6,850 persons per km^2^. The inner area as the old commercial center has many old buildings, which puts great pressure on urban renewal. The middle area was developing rapidly after the 1990s and the outer area provides a lot of cheap land for housing and infrastructure construction in the process of urban expansion.

#### Socioeconomic Variables

Socioeconomic data were obtained from the sixth Chinese population census (the year of 2010) across *jiedao* units. There are three kinds of socioeconomic variables: socioeconomic data, demographic data, and contextual data, and the descriptive statistics of these variables can be seen in Table 1. Socioeconomic data include the average level of per capita income in each unit. Demographic data include the number of population in different social groups across a range of gender groups, *hukou* groups, educational groups, and age groups. Gender groups refer to the female and male population. *Hukou* groups included local residents (Local) with *hukou* and migrants without *hukou* (Migrants). The *hukou* registration status warrants the local residents some vital services and welfare entitlements including free or subsidized health care, retirement benefits, and subsidized food and housing (25, 26), whereas migrants cannot enjoy these subsidies and social welfare as an urban citizen. Educational groups included population having middle school or below (Edu_low), high school (Edu_high), and university or above (Edu_uni). Age groups included children (under 15 years old), youths and adults(15-64) and the elder (65+). These characteristics are closely associated with high demand of urban green space (27). The contextual data mainly include population density as a rough proxy for built environment (28).

**Table 1:** Descriptive statistics of socioeconomic variables.

**Variables**	**Mean**	**Std. dev**.	**Min**	**Max**
**Gender groups**				
Female population	35,082	28,908	1,335	169,926
Male population	33,252	25,763	1,019	144,170
***Hukou*** **status**				
Local population	35,928	31,080	524	197,699
Migrants population	32,406	32,574	364	165,062
**Education background**				
Edu_low population	27,522	22,778	898	136,996
Edu_high population	16,996	10,430	474	47,587
Edu_uni population	22,125	32,422	159	22,5707
**Age groups**				
Children population	5,955	4,541	128	20,931
Youth and adult population	56,910	47,497	1,994	277,550
The elder population	5,469	3,629	105	18,580
**Socioeconomic data**				
Population density	24,581	20,972	206	90,732
Per capita income	2,215	581	481	3,954

### Methodology

#### Quantification of Urban Green Spaces

Urban green spaces are measured in two ways: mixed or woody vegetation and public parks. These two categories of urban green space reflect the different ecosystem services that urban residents may receive from different types of urban vegetation. On the one hand, mixed or woody vegetation is more related with reduced flooding, psychological benefits, biodiversity conservation, and higher air quality (9). On the other hand, public parks are more associated with opportunities for recreation and health benefits from urban health.

Previous studies mainly focus on the spatial distribution and the accessibility of various green spaces, producing the use of a “location-” or “accessibility”-based measure for green space provision (4, 29). Following the two-factor theory, distance is found to be the most important precondition for restricting the use of green space (30). Once this precondition is fulfilled, the qualities of green space (e.g., naturalness and facilities) will determine how long the users will stay (31). Thus, apart from investigating the proximity of green space, the quality of green space in relation to residents' needs will be assessed in this study.

This paper constructs the park supply index combining both the proximity and quality of green spaces, which calculated the service level of walking-distance catchments within a given spatial unit. The park supply covers by this index includes forest parks, city parks and community parks across the central city of Wuhan. The calculation of this index is detailed in Kii and Doi (27), which originated from the field of public transport. The calculation uses the following form:

(1) $$PS_{i}^{*}=\sum_{N}\frac{Area_{Bn}}{Area_{i}}\times PQ_{Bn}$$

(2) $$PS_{i}=\frac{PS_{i}^{*}-{\text{min}}_{i}PS_{i}^{*}}{{\text{max}}_{i}PS_{i}^{*}-{\text{min}}_{i}PS_{i}^{*}}$$

where *PS*_*i*_ is the normalized park supply index for the *jiedaoi* ranging 0–1;$PS_{i}^{*}$ is the park supply index for the *jiedaoi* before normalization; *N* is the number of walk access buffers to parks in each spatial unit; *Bn* denotes buffer *n* for each park in each spatial unit; *Area*_*i*_ is the spatial area of the unit *i*; *Area*_*Bn*_ is the spatial area of the buffer *n*; *PQ*_*Bn*_ denotes the park quality for each park buffer *n*, in the form of a score from 0 (the worst) to 100 (the best).

The standard for evaluating park quality combines two parameters: ecological and social. The ecological parameter includes green space proportion, lake or river, habitat animals, and habitat plants (32), whereas the social parameter includes natural beauty, recreational facilities, park size, no charge, desired visiting frequency.

**Figure 2:**
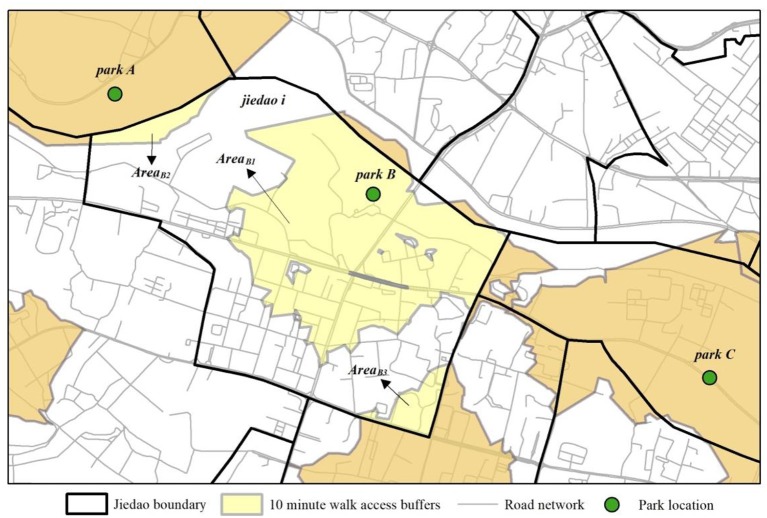
The schematic diagram how to calculate park supply index.

Figure 2 describes how to calculate this index. First, a spatial database of parks, road networks, and *jiedao*-level administrative divisions was obtained. This includes the location of park entrances, the road network, and the spatial boundary of *jiedao* units. Second, this database was integrated with a database of park quality in the central city of Wuhan. Data were calculated according to the abovementioned equation. Third, access distance to each park was measured for each *jiedao* unit assuming the following threshold of walk access, which is based on typical walk catchments (termed walk buffers) for leisure activities in parks. These are the distance that 75 or 80% of people would walk to access a park for leisure activities within 10 min of walk^1^. Fourth, the spatial analysis tools called network analysis and proximity analysis were employed in ArcGIS 10.2.

The quantity of urban vegetation is recognized to be the direct indicator reflecting the supply of green spaces. If the Normalized Difference Vegetation Index (NDVI) value of the pixel is larger than 0.1, then this pixel is classified as urban vegetation. Therein, NDVI is obtained from the WorldView-2 image with a spatial resolution of 0.60 m. The index of mixed or woody vegetation coverage ratio is calculated in this paper as below:

(3) $$\begin{array}{l} CR_{i}=\frac{GS_{i}}{Area_{i}}\times 100\% \end{array}$$

where *CR*_*i*_ denotes the coverage ratio of green space in the spatial unit *i*; *GS*_*i*_ is the total area of green space within the spatial unit *i*, which could be obtained from the NDVI imagery; *Area*_*i*_ is the total area of the particular unit *i*.

#### Theil Index for Inequity Measurement

This study deploys the Theil index for measurements of green space inequities. Theil index is applied because of its advantage in decomposability over other indicators like Gini, Atkinson, Standard Deviation, Coefficient of Variation, etc. Theil index is one special case for the Generalized Entropy family when α = 1. The calculation of Generalized Entropy and its decomposability will be elaborated as follows. Denote a population of persons, *i* = 1, …, *n*, with income *y*_*i*_. Let *f*_*i*_ = 1/*n* when the data are unweighted. Arithmetic mean income is *m*. Then, the Generalized Entropy could be formulated by

(4) $$\begin{array}{l} GE\left(1\right)=\overset{n}{\underset{i=1}{\sum }}f_{i}\times \left(y_{i}/m\right)\times log\left(y_{i}/m\right) \end{array}$$

Each *GE*(1) index can be additively decomposed as *GE*(1) = *GE*_*w*_(1) + *GE*_*b*_(1), where *GE*_*w*_(1) represents within-group inequality and *GE*_*b*_(1) denotes between-group inequality (33). Suppose there is an exhaustive partition of the population into mutually exclusive subgroups *k* = 1, …, *K*.

(5) $$\begin{array}{l} GE_{w}\left(1\right)=\overset{K}{\underset{k=1}{\sum }}S_{k}\times GE_{k}\left(1\right) \end{array}$$

where *S*_*k*_ is the share of total income held by subgroup *k*; *GE*_*k*_(1) measures the inequality for subgroup *k* and can be calculated as if the subgroup was a separate population. Meanwhile, *GE*_*b*_(1) is calculated assuming every person in a subgroup *k* obtains the mean income, *m*_*k*_.

The application of Lorenz curve and Gini index in quantifying green space can be found in the work of Wu et al. (34) measuring the inequality of effective green equivalent distribution across the urban area in Beijing. However, the Theil index is more advantageous than the Gini index because of its decomposability. This paper will employ Theil index to geographically compare green space supply in Wuhan to a broad measure of demand (different age groups, migrants, and employment distribution) and decompose this index in different regions of Wuhan (the inner, middle, and outer areas) to compare the spatial equity of urban green space.

#### The Geographic Detector Analysis

Spatial stratified heterogeneity is the spatial expression of natural and socioeconomic process, which is an important approach for human to recognize nature. Geographic detector analysis is a new statistical method to detect spatial stratified heterogeneity and reveal the driving factors behind it. The study area is characterized by spatial stratified heterogeneity if the sum of the variance of subareas is less than the regional total variance, and if the spatial distribution of the two variables tends to be consistent, there is statistical correlation between them. *Q*-statistic in geographic detector analysis has been widely applied in many fields of natural and social sciences.

Here, the geographic detector analysis is utilized to detect spatial stratified heterogeneity between urban green spaces and socio-economic variables. The determinant power of covariate *X* to the spatial pattern of urban green spaces, or the *q* statistics, is defined as follows:

(6) $$\begin{array}{l} q=1-\frac{SSW}{SST} \end{array}$$

(7) $$\begin{array}{l} SSW=\sum_{h=1}^{L}N_{h}\sigma_{h}^{2},\;SST=N\sigma^{2} \end{array}$$

where *h* = 1, 2, …, *L* is the strata of variable *Y* or covariate *X*; *N*_*h*_ is the number of strata *h*; *N* is the number of spatial units; $\sigma_{h}^{2}$ and σ^2^, respectively, refer to the variance of *Y* in strata *h* and the whole region. *SSW* is the within sum of squares whereas *SST* is the total sum of squares. The *q* statistics ranges between 0 and 1. A larger value of *q* statistics indicates the better explaining power of covariate *X* on the distribution of *Y*.

## Results

### Horizontal Equity Across All Population

Table 2 demonstrates the decomposable Theil index of various green spaces in Wuhan. Theil coefficients of PS and CR were 0.29 and 0.09, demonstrating that the overall supply of public parks is far more unequal across *jiedao*s of Wuhan than urban vegetation coverage. Residents in Wuhan tend to equally enjoy the psychological and biodiversity benefits from the mixed and woody vegetation. However, residents in Wuhan cannot enjoy equally the recreation and the related health benefits from the parks, mainly depending on their socio-economic status.

**Table 2:** The Theil index of various green spaces in the inner, middle, and outer areas of Wuhan.

**Type**	**PS**	**CR**
Overall	0.29	0.09
Between group	0.05	0.02
Within group	0.24	0.07
Inner area	0.17	0.12
Middle area	0.37	0.03
Outer area	0.50	0.04

*PS denotes park supply index; CR means the coverage ratio of urban vegetation*.

To detect the influence of spatial circular structure on the provision of green spaces, we decompose the overall Theil index across *jiedao*s by three groups, namely, Inner area, Middle area, and Outer area. As seen in Table 2, the Theil index of PS and CR within groups arrives at 0.24 and 0.07, whereas the Theil index of PS and CR between groups is only 0.05 and 0.02. Therefore, the inequitable distribution of parks as well as mixed and woody vegetation is mainly demonstrated within the inner, middle, and outer areas.

Moreover, the Theil indices of PS within the Inner area, Middle area, and Outer area are 0.17, 0.37, and 0.50, respectively. The distribution of public parks is more inequitable in the outer area than both the inner and middle areas. Residents living in the urban core are more likely to enjoy the recreation and health benefits from public parks where residents living in the urban suburb are unable to enjoy the corresponding benefits, depending on their socio-economic status. Meanwhile, the Theil indices of CR within the Inner area, Middle area, and Outer area are 0.12, 0.03, and 0.04. The distribution of mixed and woody vegetation is relatively equitable in the Inner, Middle, and Outer areas.

Due to the ongoing housing reform and the massive process of urban sprawl, residential spaces in urban China are changing from largely mixed work-unit compounds toward differentiated commercial neighborhoods (35). Due to historical reasons of urban development in China, the work-unit compounds were equipped without landscape greening and they are mainly distributed in the urban core. The local government was responsible for constructing parks to satisfy residents' demand for leisure activities. Thus, the park supply in the inner area is relatively equitable. However, as a result of housing reforms and marketization, the commercial neighborhoods provided by the real estate developers, are equipped with good greening environment and are mainly distributed in the middle areas and urban suburbs. Therefore, the number of parks constructed by the municipal government is only a few, resulting in the largely unequal distribution of parks especially in urban suburbs and exurban zones.

### Mapping Horizontal Equity Gaps

**Figure 3:**
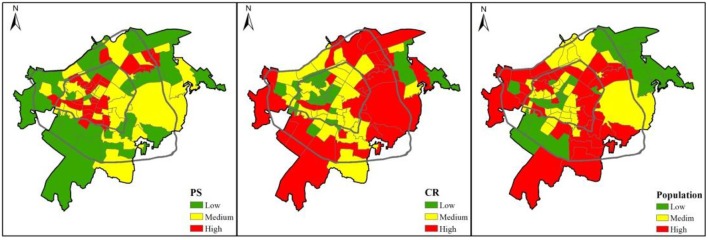
The spatial pattern of PS, CR, and population in Wuhan.

Figure 3 demonstrates the spatial pattern of PS, CR, and population in Wuhan. Overall, the Inner area has higher PS, indicating that more public parks are concentrated in the city core. However, the middle and outer areas have higher CR, whereas the inner area is relatively lacking of mixed or woody vegetation. The distribution of population also demonstrates significant spatial variations. More population are clustered in the educational and high-tech town of Wuchang because many universities and high-tech enterprises are situated there to attract many college students and employees in the field of information and computer technology.

Figure 3 presents the distributions of *jiedao* units by population, PS, and CR quartiles. 34.83% of spatial units have high population yet low PS whereas 37.05% of spatial units have low population yet high PS, indicating that almost 40% of *jiedao*s in Wuhan suffer from the shortage of public parks. Moreover, 32.58% of spatial units have high population yet low CR whereas 24.72% of spatial units have low population yet high CR, indicating that more than 30% of *jiedao*s in Wuhan encounter the shortage of mixed or woody vegetation.

**Figure 4:**
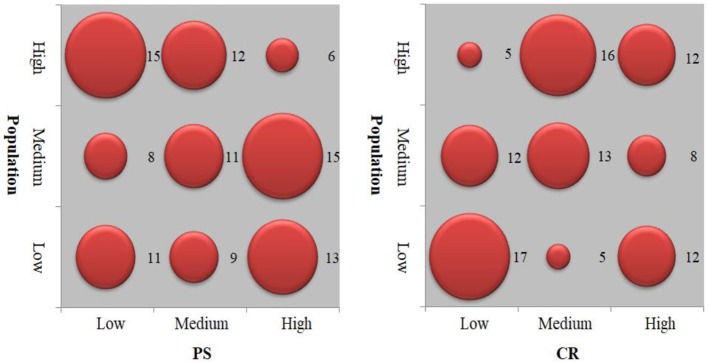
Percentage of *jiedao* units in each population, PS, and CR quartiles. Values in each panel total 100%.

Figure 4 also illustrates spatial units in the most and least desirable quartile for each attribute: “Sweet-spot” spatial units have a high supply of green spaces and have a low population; “sour-spot” spatial units are the opposite (low green spaces, high population). We observed from Figure 2 that the sweet-spot neighborhoods are mainly distributed in the old work-unit compounds nearby the location of the provincial and municipal governments in the inner area. On the one hand, these sweet-spot neighborhoods have easier accessibility to nearby public parks and the street sides were planted with woody trees having been growing for decades. On the other hand, as the old resident quarters, these sweet-spot neighborhoods are supplied with aging municipal public infrastructure and lack of public services. Since 2016, the Chinese government has paid attention to urban redevelopment and advanced some reform policies to the reconstruction of these old residential quarters.

On the contrary, the sour-spot neighborhoods are mainly distributed in the newly developed commercial residences in the middle or outer areas. These sour-spot neighborhoods are mainly equipped with private gardens within the gated communities provided by estate developers. Usually, high-income residents are more likely to enjoy better greening environment and the corresponding benefits.

### The Spatial Relation Between Socioeconomic Contexts and Urban Green Spaces

The geographic detector tool is employed to examine how socioeconomic contexts and social groups have influence on the distribution of public parks and mixed or woody vegetation. As seen in Table 3, the proportions of different social groups have differing explaining power on the distribution of public parks. First, the influence of education on the distribution of public parks is statistically significant at the 1% level. The spatial heterogeneity of education has more than 20% of explaining power on the spatially differentiated distribution of public parks. Therein, Prop Edu_mid has the largest explaining power (30%) on the distribution of public parks. Second, the influence of gender on the distribution of public parks is not statistically significant. Third, Prop Age below_15 and Prop Age above 65 have, respectively, 28 and 27% of explaining power on the distribution of public parks. This indicates that families with teenagers or elder people tend to reside in nearby public parks. Fourth, the importance of *hukou* is not statistically significant for explaining the distribution of public parks. Neither local residents nor migrants have preferences on living nearby public parks to enjoy the health benefits. However, the influence of different social groups on mixed or woody vegetation is not statistically significant.

**Table 3:** The *q* statistics between urban green spaces and different population groups.

**Type**	**Variables**	**PS**	***P*-value**	**CR**	***P*-value**
Education	Prop Edu_low	0.21	0.05	0.24	0.79
	Prop Edu_high	0.30	0.03	0.25	0.36
	Prop Edu_uni	0.20	0.08	0.21	0.37
Gender	Prop female	0.15	0.51	0.10	0.99
	Prop male	0.18	0.27	0.08	0.97
Age	Prop age below_15	0.28	0.01	0.20	0.69
	Prop age 16–64	0.16	0.18	0.23	0.53
	Prop age above 65	0.27	0.01	0.16	0.65
*Hukou*	Prop local	0.14	0.41	0.13	0.82
	Prop migrants	0.14	0.39	0.14	0.83
Population density	0.31	0.01	0.47	0.00	
Per capita income	0.26	0.01	0.13	0.76	

*PS denotes park supply index; CR means the coverage ratio of urban vegetation*.

Population density, as a proxy for the built environment, also clearly plays a role in the distribution of both public parks and urban vegetation. The spatial pattern of population density, respectively explains 31% of the distribution of public parks and 47% of the distribution of urban vegetation. This supports the theory that urban green spaces originate from the social need to improve their living environment. On the one hand, the government tends to build more public parks and plant more urban vegetation in densely populated areas to satisfy residents' need for better greening environment. On the other hand, the neighborhoods with well-provided public parks and urban vegetation become attractive and then draw more residents to live in this neighborhood.

Per capita income is statistically significant and could explain 26% of the distribution of public parks. However, per capita income is not significantly associated with mixed and woody vegetation. This proves that people with higher income tend to live nearby public parks and enjoy the recreation and health benefits. The positive association between per capita income and the supply of parks indicates that the greening of neighborhoods may cause and/or enhance gentrification, which is a social equity problem because it pushes out low-income residents in favor of high-income in-migrants (36). When green gentrification follows, it leads to increased concentrations of wealthy people living along the parks.

### Vertical Equity for Different Social Groups

**Figure 5:**
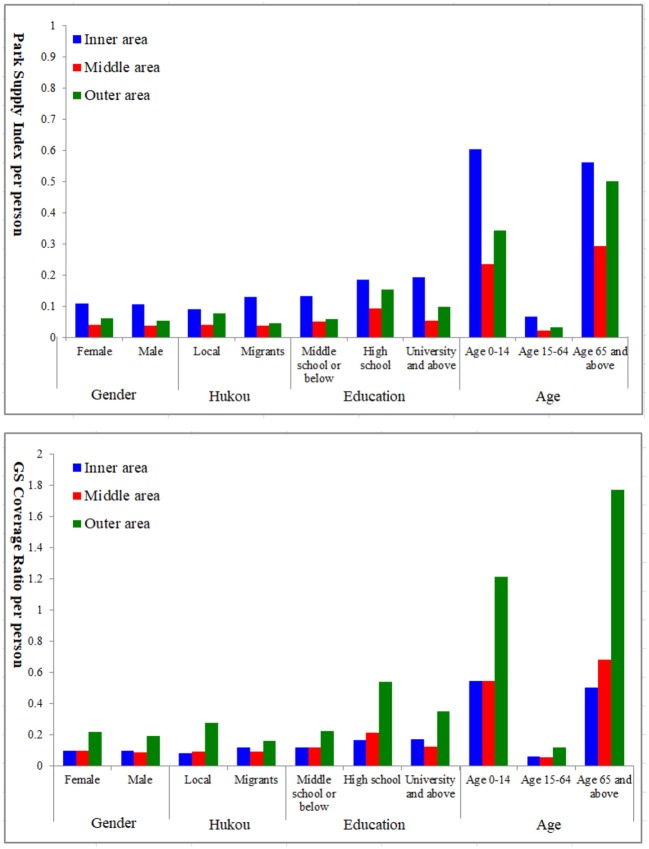
Vertical equity of urban green spaces.

As demonstrated in Figure 5, urban green space supply is highly skewed toward the inner and outer areas. Regarding the park supply, all social groups in the inner area enjoy more green space supply than the other two areas. This suggests some vertical equity “advantage” of park supply for vulnerable social groups in the urban core. Regarding the urban vegetation supply, the urban outer area is equipped with more green space to all social groups than other areas, implying a degree of vertical equity “advantage” in the urban suburb.

Table 4 demonstrates the Theil indices for vulnerable groups in the inner, middle, and outer areas. High values of Theil indices indicate the highly inequitable supply within three regions of Wuhan for each vulnerable group. Therein, regarding the park supply, the inner area has the highest Theil index for females, residents with low education, children, and the elder whereas the outer area has the highest Theil index for the migrants. Regarding the coverage rate of urban vegetation, the outer area has the highest Theil index for all vulnerable groups. This indicates that unequal recreation and health benefits are provided for these vulnerable groups especially in the inner area, whereas these vulnerable groups get unequal access to mixed and woody vegetation to enjoy the psychological and biodiversity benefits especially in the outer area.

**Table 4:** Theil indices for vulnerable groups in the inner, middle, and outer areas.

**Type**	**PS**			**CR**		
	**Inner**	**Middle**	**Outer**	**Inner**	**Middle**	**Outer**
Female	0.85	0.47	0.84	0.46	0.23	0.74
Migrants	0.50	0.65	0.86	0.33	0.34	1.26
Edu_low	0.82	0.47	0.76	0.55	0.42	0.67
Children	1.03	0.46	0.72	0.58	0.39	0.64
The elder	1.29	0.44	0.44	0.71	0.35	0.77

*PS denotes park supply index; CR means the coverage ratio of urban vegetation*.

The inequitable supply of urban green spaces for vulnerable groups especially in the inner and outer areas can be attributed in the following two aspects: first, the greening initiatives in the inner area, such as renovation of old residential areas and constructing pocket parks contribute to environmental sustainability and economic sustainability, but not social sustainability. The greening of neighborhoods prepares them for gentrification, which allows elites (politicians and real estate developers) to benefit (36). Second, the coalition of real-estate developers and political elites transforms the brownfields in the outer area into residential neighborhoods with good environmental amenities. Through real-estate development associated with the greening, the green growth machine turned a profit and demographically transformed neighborhood through this process of green gentrification (37).

## Discussions

The inequity of urban green spaces is mainly demonstrated in terms of public parks instead of mixed or woody vegetation. This finding also highlights that the ecosystem services provided by urban vegetation are more equitably distributed, with recreational benefits provided by public parks being differentially distributed. This finding is in contrast to the empirical evidence on 10 U.S. cities that parks are more equitably distributed and that the increased inequity observed in the mixed and woody vegetation may be due to vegetation located on private land or streets (9). However, in the background of the socialist marketization with Chinese characteristics, urban green spaces as a public product are mainly planned and managed by the local government, which prefers to plant more trees instead of constructing parks.

Through the decomposable Theil index, the circular spatial structure has significant influence on the equity of urban green spaces. Therein, the distribution of public parks is more inequitable in the outer area, whereas the mixed and woody vegetation is more spatially differentiated in the inner area. The spatial equity of urban green spaces are closely associated with the ongoing housing reforms toward marketization and the massive process of urban sprawl, which may further enhance the inequity of greening environment in the urban China (17). Usually, high-income residents tend to live in the neighborhoods with private gardens within the gated communities provided by estate developers and enjoy better greening environment as well as corresponding benefits.

This paper confirms that there is widespread evidence of green inequity, supporting theories of environmental justice and political ecology that suggest that environmental amenities are inequitably distributed across different social groups (9, 17). Neighborhoods with education above high school and more proportion of children and elder people are often associated with increased access to parks. Younger dwellers tend to prefer ecosystem services facilitating social interactions whereas older inhabitants prefer ecosystem services related to nature experiences (38). These diverging perceptions should be taken into account through urban development strategies to create a socially just and sustainable city in the face of global environmental changes.

Per capita income clearly plays a role in the distribution of parks, suggesting that environmental amenities are inequitably low in communities with lower economic power (39). Higher incomes are often associated with increased access to resources in society, while lower incomes are often associated with deprivation (28). Environmental amenities draw in wealthier groups of residents and push out lower-income residents, thus creating gentrification (36). Policy interventions are required in China to reduce environmental inequality brought by urban greening.

Although there is some evidence of vertical equity advantages for vulnerable groups with more parks supply in the inner area and more urban vegetation in the outer area, the highly internal differences of urban green spaces within the inner, middle, and outer areas should be noted. These vulnerable groups tend to gravitate toward different types of housing and location, leading to differing opportunities to enjoy the corresponding health benefits. How to incorporate the demand of vulnerable groups in the planning of green infrastructure becomes important to promote social justice and urban sustainability.

## Conclusions and Policy Implications

This paper aims to enrich the empirical analyses of green space equity with a case study of an inland city of China. Two contrasting perspectives are compared to examine the horizontal and vertical equity of public parks and urban vegetation. The decomposable Theil index is employed to investigate the influence of circular spatial structure on the equity of green spaces. Through mapping horizontal equity gaps, we can observe the sweet spot and the sour spot of green spaces. Then, the geographic detector analysis is utilized to examine their spatial relation between socioeconomic contexts and urban green spaces. The following conclusions can be drawn:

First, the inequity of public parks is more intense than mixed and woody vegetation. The influence of circular spatial structure on the distribution of parks is inevitable that the outer area has higher inequity of the park supply than the inner and middle areas. Second, socioeconomic variables, such as education, age, population density, and per capita income demonstrate significant impact on the distribution of parks. However, only population density shows close association with the distribution of mixed and woody vegetation. Third, urban green space supply is highly skewed toward the inner and outer areas. Vulnerable groups, such as females, residents with low education, migrants, children, and the elder have inequitable opportunities to enjoy the health benefits of parks and vegetation especially in the inner and outer areas.

The above findings shed some important insights into urban sustainability and promoting social and environmental justice. The inequitable distribution of green spaces implies that policy interventions are required to reduce environmental inequality. The spatial factor is important to guide the spatial equity of green spaces that the outer area has more shortage of public parks than the inner and middle areas. Micro-sized green spaces, such as pocket parks are recommended in urban suburbs to improve residents' access to green spaces. The social factor is also significant for urban planners and policy-makers to promote environmental justice. This paper confirms that environmental amenities are inequitably distributed across different social groups. The diverging perceptions of various social groups should be taken into account through urban development strategies to create a socially just and sustainable city in the face of global environmental changes. Furthermore, green gentrification in urban China may enhance the inequity of greening environment. The top-down process of green space management in China tends to allow elites and politicians to enjoy the green benefits. Creative governance of urban green space, such as shared governance involving multiple stakeholders is proposed to provide a cooperative atmosphere for decision making and active citizenship through volunteering.

There are two limitations of this study. The dimension of equity includes equity of opportunity and equity of outcome. This paper mainly focuses on the former but ignores the latter. Further studies could compare the differences between equity of opportunity and equity of outcome for urban green spaces. Moreover, this paper presents a novel method combining proximity and quality to measure the spatial supply of urban parks. The advantage of the proposed method in this paper is its relative simplicity to assess the spatial supply of public parks although the calculation of the park supply index is somewhat limited since it does not measure the access to specific destinations and only considers the walk catchments.

## Data Availability Statement

The datasets analyzed in this article are not publicly available. Requests to access the datasets should be directed to sanwei.87@163.com.

## Author Contributions

SH: designing the manuscript, and structure and writing the manuscript. YW: data processing and map designing. LW: indicator calculation and result analysis.

### Conflict of Interest

The authors declare that the research was conducted in the absence of any commercial or financial relationships that could be construed as a potential conflict of interest.

## Footnotes

^1^According to the study of Nesbitt et al. (9), many municipalities in U.S. use a 10-min walk to a park as a park accessibility target.
